# Editorial: Big data shaping clinical trial landscape—Greater role for pharmacoeconomics in Asia

**DOI:** 10.3389/fpubh.2022.1037082

**Published:** 2022-10-26

**Authors:** Mihajlo Jakovljevic, Kevin Lu

**Affiliations:** ^1^Institute of Advanced Manufacturing Technologies, Peter the Great St. Petersburg Polytechnic University, St. Petersburg, Russia; ^2^Institute of Comparative Economic Studies, Hosei University, Tokyo, Japan; ^3^Department of Global Health Economics and Policy, University of Kragujevac, Kragujevac, Serbia; ^4^University of South Carolina College of Pharmacy, Columbia, SC, United States

**Keywords:** clinical trial, pharmacoeconomics, Asia-Pacific, APAC, regulatory framework, market access

Big Data generated across diverse fields of clinical medicine are increasingly becoming accessible across some of the most rapidly developing world regions. This is particularly the fact with the Asia-Pacific region of Asia or “APAC”. It is now positioned as the second largest global market for pharmaceuticals and medical devices, behind North America while overrunning the pace of development in Western European EU5 pharmaceutical markets. The competitiveness of multinational pharmaceutical manufacturers based in this region is documented by the recent 2021 ranking of four Chinese companies (Sinopharm, Guangzhou Pharmaceuticals Corporation, Shanghai Pharmaceutical, and Yunnan Baiyao) among the Top 25, per their annual net revenues. Long-term forecasts predict that pharmaceutical expenditure will continue to rise faster than real GDP growth across the APAC region (1). Population aging is entering an advanced stage which increases the likelihood of an increased number of middle-class citizens. Their purchasing power contributes to the strong demand for innovative pharmaceuticals. Many of these cutting-edge medicines ranging from monoclonal antibodies to targeted oncology are blockbuster drugs leading to ultimate budget impact and prohibitively expensive reimbursement prices.

This phenomenon has forced Japanese authorities to explore the Korean Health Technology Assessment's historical legislation and introduce cost-effectiveness-based resource allocation and annual price cuts (2). Similar measures at a large scale are currently being undertaken in mainland China to tackle the drug bill and impose cost containment. Diverse reimbursement approaches and comprehensive insurance coverage is extended, covering an increasing share of Chinese suburban and rural populations. A similar chain of events is now taking place across ASEAN countries. National authorities across APAC, primarily Ministries of Health, Social Insurance Funds, and Medicines Approval Agencies are joining forces to adopt an improved legislative framework for the upcoming 2020s. Pharmacoeconomics' analytical capacities and an understanding of cost-effectiveness-based resource allocation are gaining a firmer foothold in the policymakers' mindset across Western Pacific.

All of these profound changes imply the acceleration of the widespread understanding of Big Data sets and the adoption of theory and practice of Pharmacoeconomics and Value-based healthcare. There was an array of contributors to this topic who closely elaborated on challenges and opportunities within this field of knowledge.

Lou et al. have contributed a study entitled: “Sacubitril-valsartan for the treatment of hypertension in China: A cost-utility analysis based on a meta-analysis of randomized controlled trials”. This study compares two highly efficient antihypertensive agents sacubitril-valsartan vs. angiotensin receptor blockers or placebo. Their findings conclude that sacubitril-valsartan is more effective than valsartan in reducing blood pressure and provides more quality-adjusted life-years (QALYs), although with higher costs. Sacubitril-valsartan is cost-effective for hypertension in contemporary Chinese circumstances under the willingness-to-pay threshold of three times per capita GDP (Lou et al.).

Zhao et al. have studied the effect of the coronavirus pandemic while using a hybrid model architecture for analyzing and optimizing COVID-19 data throughout all of the provinces of mainland China. A variety of advanced analytical methods such as Logistic regression (LR), Autoregressive Integrated Moving Average Model (ARIMA), support vector regression (SVR), multilayer perceptron (MLP), Recurrent Neural Networks (RNN), Gate Recurrent Unit (GRU), and long short-term memory (LSTM) were exploited for this purpose. The authors report that the number of quarantines, mortality rate, and the deployment of public self-protection measures to reduce the epidemic were carefully measured. They believe such projections may be used by governmental authorities to guide future illness prevention and control health policy choices (Zhao et al.).

Another Chinese research group worked on an intriguing health econometric comparison, estimating the impact of donafenib's and sorafenib's cost-effectiveness when both are indicated as the first-line treatment of unresectable or metastatic hepatocellular carcinoma. In a methodologically stringent framework applied to Chinese patients (a three-state partitioned survival model) they have proven that donafenib is unlikely to be cost-effective compared with sorafenib in this indication field. A plausible price reduction strategy applied to the market penetration of donafenib might increase its cost-effectiveness in the future evolving market landscape in China (Meng et al.).

A mixed Pakistani- Saudi research group has brought our attention to interesting methodological challenges in statistics applied to clinical oncology. Namely, the authors attempt to improve the variance estimator of the Kaplan–Meier survival function, in a mathematical setting with zero, moderate, and heavy censoring. The authors propose an innovative modified cumulative hazard function, the plot of which provides far more detailed insight in the case of heavy censoring (Khan et al.).

Summing up, the array of aforementioned contributions alongside this conclusive Editorial article point to the rapidly increasing role of Big Data and their advanced analytics in the eastern and western Asia regions. South-East Asia presents a hotbed of innovation strategies in artificial intelligence and the fourth industrial revolution, led by Chinese think tanks. Thus, it means that the administration of such data sets and their AI-assisted analytical opportunity in the healthcare arena might present distinctive room for the opportunity of further development (3). This is also largely visible when considering the market landscape in medical devices in ASEAN countries and the broader South Asia region as witnessed by a recent Nepalese study (4). Furthermore, the ability of Asian OECD and LMIC societies to invest in Big Data related developments is largely conditional on their overall R&D spending and health expenditures (1, 5). Mammoth-sized Chinese private sector companies have already entered a struggle to survive and strive in Big Data competition (6, 7). Xiaomin et al. have pointed out to a great extent how ongoing Big Data legislation dynamics are highly likely to reshape the entire dynamic of marketing approvals and the reimbursement of pharmaceuticals and medical technologies (8). Yet to what extent this accelerating development evolves in years to come remains to be seen (9).

## Author contributions

MJ prepared the manuscript draft. KL revised it for important intellectual content. All authors contributed to the article and approved the submitted version.

## Funding

This study has been co-financed through the Grant OI 175 014 of the Ministry of Education Science and Technological Development of the Republic of Serbia.

## Conflict of interest

The authors declare that the research was conducted in the absence of any commercial or financial relationships that could be construed as a potential conflict of interest.

## Publisher's note

All claims expressed in this article are solely those of the authors and do not necessarily represent those of their affiliated organizations, or those of the publisher, the editors and the reviewers. Any product that may be evaluated in this article, or claim that may be made by its manufacturer, is not guaranteed or endorsed by the publisher.

## References

[1] Jakovljevic M Timofeyev Y Ranabhat C. Real GDP growth rates and healthcare spending – comparison between the G7 and the EM7 countries. Global Health. (2020) 16:64. 10.1186/s12992-020-00590-3 32677998 PMC7367257

[2] Ogura S Jakovljević M. Health financing constrained by population aging: an opportunity to learn from Japanese experience. Serb J Exp Clin Res. (2014) 15:175–81. 10.2478/sjecr-2014-0022

[3] Jakovljevic M. Therapeutic innovations: the future of health economics and outcomes research–increasing role of the Asia-Pacific. J Med Econ. (2021) 24(sup1:i–iii.) 10.1080/13696998.2021.2014164 34859736

[4] Sapkota B Palaian S Shrestha S Ozaki A Mohamed Ibrahim MI Jakovljevic M. Gap analysis in manufacturing, innovation and marketing of medical devices in the Asia-Pacific region. Exp Rev Pharmacoecon Outcomes Res. (2022). 10.1080/14737167.2022.2086122 35658768

[5] Jakovljevic M Sugahara T Timofeyev Y Rancic N. Predictors of (in) efficiencies of healthcare expenditure among the leading Asian economies–comparison of OECD and Non-OECD Nations. Risk Manag Healthc Policy. (2020) 13:2261–80. 10.2147/RMHP.S266386 33117004 PMC7585857

[6] Shamim S Zeng J Shariq SM Khan Z. Role of big data management in enhancing big data decision-making capability and quality among Chinese firms: a dynamic capabilities view. Inf Manag. (2019) 56:103135. 10.1016/j.im.2018.12.003

[7] Saleem H Li Y Ali Z Mehreen A Mansoor MS. An empirical investigation on how big data analytics influence China SMEs performance: do product and process innovation matter? In: Corporate Performance and Managerial Ties in China. Routledge (2021). p. 9–34.

[8] Xiaomin D Yan L Lihua S. Impact of big medical data on medical record data formation model from pharmacoeconomic evaluation perspectives. 亚洲社会药学. (2016) 11:112–5. 10.1080/13602381.2020.1759300

[9] Yin T. Application research of precision medical model based on big data in primary health care in China. Afr Asian Stud. (2020) 19:323–35. 10.1163/15692108-12341470

